# Optical frequency synthesizer with an integrated erbium tunable laser

**DOI:** 10.1038/s41377-019-0233-z

**Published:** 2019-12-18

**Authors:** Ming Xin, Nanxi Li, Neetesh Singh, Alfonso Ruocco, Zhan Su, Emir Salih Magden, Jelena Notaros, Diedrik Vermeulen, Erich P. Ippen, Michael R. Watts, Franz X. Kärtner

**Affiliations:** 1 Research Laboratory of Electronics, Massachusetts Institute of Technology, Cambridge, MA 02139 USA; 2 John A. Paulson School of Engineering and Applied Science, Harvard University, Cambridge, MA 02138 USA; 3 Center for Free-Electron Laser Science, DESY and Hamburg University, Notkestraße 85, 22607 Hamburg, Germany; 4 Present Address: Institute of Microelectronics, Agency for Science, Technology and Research (A*STAR), 138634 Singapore, Singapore; 5 Present Address: Analog Photonics, 1 Marina Park Drive, Boston, MA 02210 USA; 6 Present Address: Department of Electrical and Electronics Engineering, Koç University, Sarıyer, Istanbul, 34450 Turkey

**Keywords:** Frequency combs, Integrated optics

## Abstract

Optical frequency synthesizers have widespread applications in optical spectroscopy, frequency metrology, and many other fields. However, their applicability is currently limited by size, cost, and power consumption. Silicon photonics technology, which is compatible with complementary-metal-oxide-semiconductor fabrication processes, provides a low-cost, compact size, lightweight, and low-power-consumption solution. In this work, we demonstrate an optical frequency synthesizer using a fully integrated silicon-based tunable laser. The synthesizer can be self-calibrated by tuning the repetition rate of the internal mode-locked laser. A 20 nm tuning range from 1544 to 1564 nm is achieved with ~10^−13^ frequency instability at 10 s averaging time. Its flexibility and fast reconfigurability are also demonstrated by fine tuning the synthesizer and generating arbitrary specified patterns over time-frequency coordinates. This work promotes the frequency stability of silicon-based integrated tunable lasers and paves the way toward chip-scale low-cost optical frequency synthesizers.

## Photonics: Highly tunable optical frequency synthesizer for on-chip applications

A tiny synthesizer capable of producing finely tuned laser light could prove invaluable in photonics applications. Radio frequency synthesizers, which have long been used in communications technologies, generate a range of frequencies from a single input. The ability to fine-tune light frequencies in the same way could transform data transmission and precision navigation devices, but the size, cost and power consumption of existing optical frequency synthesizer (OFS) designs make them impractical for applications outside scientific laboratories. Now, Ming Xin, Nanxi Li at Massachusetts Institute of Technology, Cambridge, United States, and the co-workers have developed an OFS that is fully compatible with CMOS technology, which has the potential to radically reduce the volume and cost of OFSs. Their OFS uses an integrated laser doped with erbium, enabling wide tunability across a broad wavelength range.

## Introduction

In the past two decades, there has been much research and development in optical frequency synthesizers (OFSs), which are able to generate accurate and stable optical frequencies from a single microwave frequency reference. OFSs have enabled a wide range of applications in frequency metrology^1–3^, precise navigation^4–6^, optical spectroscopy^7–9^, microwave photonics^10–12^, and so on. However, applications are often limited to scientific experiments due to the size, weight, power consumption, and cost of OFSs. To improve their applicability, OFSs based on chip-scale-integrated photonic devices have been proposed and demonstrated^13–17^. Among different kinds of integrated photonics platforms, silicon photonics technology has been extensively developed to meet the increasing demand for data communication bandwidth. Since it is compatible with the mature complementary-metal-oxide-semiconductor (CMOS) fabrication technology, this technology can cost-effectively mass-produce chip-scale devices on the wafer level. Therefore, silicon photonics is a promising candidate to radically reduce the volume and cost of OFSs and eventually bring all the benefits of OFSs from the laboratory to our daily lives.

A tunable laser (TL) is one of the most important elements in an OFS since it acts as the source of the final output optical signal of the synthesizer. Recently, rare-earth-doped integrated lasers have been developed on silicon photonics platforms. These kinds of lasers possess several advantages that make them suitable for chip-scale OFSs. First, the gain media can be deposited through a single-step back-end-of-line process, enabling CMOS-compatible monolithic laser integration for low-cost mass production^18^. Common rare-earth elements such as erbium, thulium, and holmium have broad gain bandwidths, which enable wide tunability over different wavelength regions^19–23^. Furthermore, rare-earth-doped integrated lasers can achieve narrow linewidths since these materials do not involve free carriers in the pumping process^24–26^. In addition, the host material of the gain elements has low thermo-optic coefficients and hence enables lasers with good thermal stability^27,28^. Although the performance of these silicon photonics-integrated lasers has been fully characterized on the device level, it is still unknown if they are qualified for OFSs, which need much more stringent requirements than free-running integrated lasers in terms of frequency instability, tuning precision, reconfigurability, and so on.

To this matter, an OFS using a silicon photonics-based integrated TL is demonstrated in this work. By carefully designing the tuning mechanism and control electronics, a large tuning range with precise and flexible tuning, high frequency stability and self-frequency calibration is achieved simultaneously. Therefore, the uncertainty associated with integrated TLs, which is one of the largest technical risks that prohibit the realization of chip-scale OFSs, is eliminated.

## Results

### Synthesizer setup

**Fig. 1:**
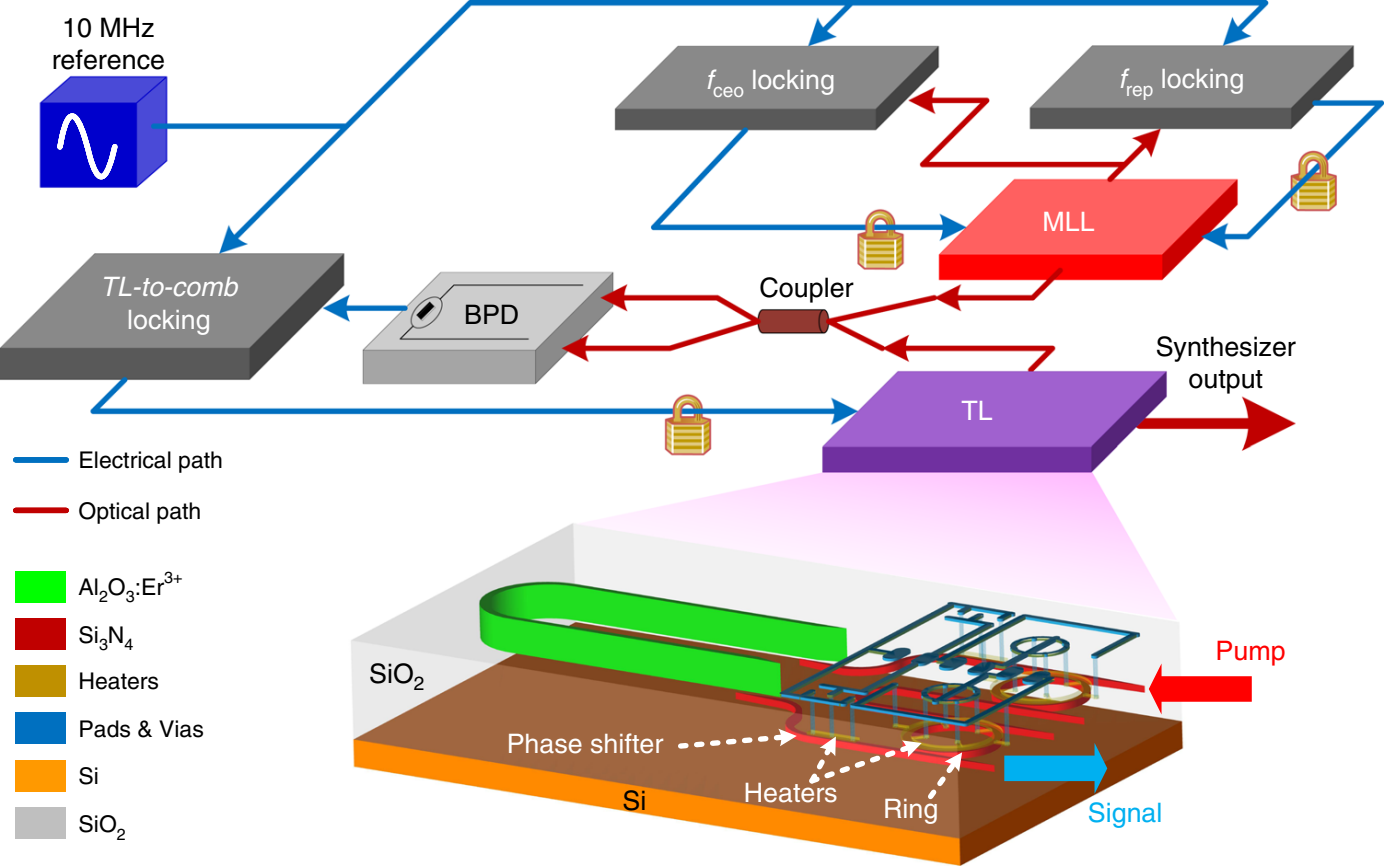
Top: the architecture of an OFS. Both the carrier offset frequency, *f*_ceo_, and the repetition rate, *f*_rep_, of a mode-locked laser (MLL) are referenced to a 10 MHz reference signal through an *f*_ceo_ locking block and an *f*_rep_ locking block. The output of the MLL and a TL are combined using a 50:50 fiber coupler and beaten in a balanced photodetector (BPD). The output of the BPD is sent to the TL-to-comb locking block, which also references the 10 MHz signal. The TL-to-comb locking output is sent to the TL to lock the frequency of TL to one comb line of the MLL. **Bottom: schematic of the TL (not to scale)**. The laser cavity is located in the Al_2_O_3_:Er^3+^ layer and the Si_3_N_4_ layer. A >4-cm-long bent gain waveguide in the Al_2_O_3_:Er^3+^ layer is used to provide sufficient gain. In the Si_3_N_4_ layer, there are two microring filters in a Vernier configuration and two longitudinal-mode phase shifters, which are controlled by the metal heaters on their top layer, to tune the wavelength of the TL.

**Fig. 2:**
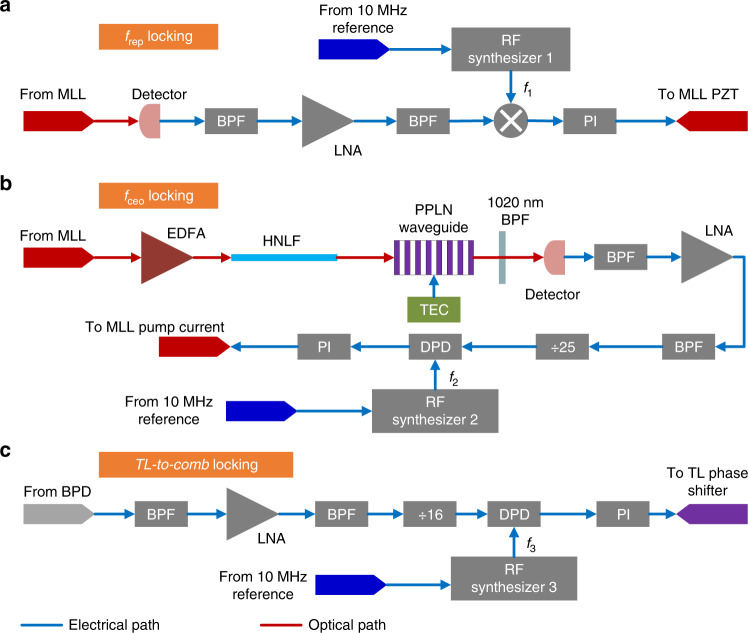
The locking circuits of the OFS. **a** In the *f*_rep_ locking circuit, the output of the MLL, whose *f*_rep_ is close to 250 MHz, is first detected by a detector; the fourth harmonic of *f*_rep_ is filtered out by a bandpass filter (BPF), amplified by a low-noise amplifier (LNA) and filtered again by another BPF. Then, the amplified and cleaned 4*f*_rep_ signal is mixed with another RF signal at frequency *f*_1_ from RF synthesizer 1. The output of the mixer is sent to a proportional integral (PI) controller to generate an error signal, which is fed back to a piezoelectric actuator (PZT), to lock *f*_rep_ to *f*_1_/4. **b** In the *f*_ceo_ locking circuit, the output of the MLL is amplified by an erbium-doped fiber amplifier (EDFA) and goes through a highly nonlinear fiber to generate a supercontinuum spectrum from 1000 to 2100 nm. The supercontinuum signal is launched into a periodically poled lithium niobate (PPLN) waveguide to double the frequency component from ~2040 to 1020 nm. The temperature of the PPLN is stabilized to 160 °C by a temperature controller (TEC). The original and new generated 1020 nm signals are filtered out by an optical BPF and beaten in a detector to obtain *f*_ceo_, which is amplified and cleaned by an LNA and two BPFs. Then, the signal is divided by 25 and compared with *f*_2_ from RF synthesizer 2 in a digital phase detector (DPD). The output of the DPD is sent to a PI controller to generate an error signal, which is fed back to control the pump current of the MLL, to lock *f*_ceo_ to 25*f*_2_. **c** In the TL-to-comb locking circuit, the beat note of the MLL and the TL is first amplified and cleaned by an LNA and two BPFs; then, it is divided by 16 and compared with *f*_3_ from RF synthesizer 3 in a DPD. The output of the DPD is fed back through a PI controller to control the phase shifter of the TL to lock the frequency of the TL to one comb line of the MLL with an offset frequency of 16*f*_3_.

The architecture of an OFS is shown in Fig. 1. It consists of an integrated erbium TL^29^ and a mode-locked laser (MLL). We choose a commercial MLL so that we can focus on the technical limitations of the integrated TL device in an OFS system. After stabilizing the *f*_ceo_ and *f*_rep_ of the MLL and locking the frequency of the TL to one comb line of the MLL, the output of the TL can serve as the synthesizer output. The detailed circuits of the three locking blocks, *f*_ceo_ locking, *f*_rep_ locking, and TL-to-comb locking, are shown in Fig. 2a–c, respectively. Based on these locking electronics, the synthesizer output frequency is given by:

1 $$f_{\mathrm{SN}} = \frac{1}{4}Mf_1 - {\mathrm{25}}f_2 - 16f_3$$

where *M* is the comb line mode number of the MLL and *f*_1_, *f*_2_, and *f*_3_ are the output frequencies of RF synthesizers 1, 2, and 3 in Fig. 2a–c, respectively. Since RF synthesizers 1, 2, and 3 are referenced to the same 10 MHz signal, the RF frequency (10 MHz) stability is transferred to the optical frequency *f*_SN_. This frequency stability transfer is the fundamental purpose of building an OFS.

### Mode number calibration

**Fig. 3:**
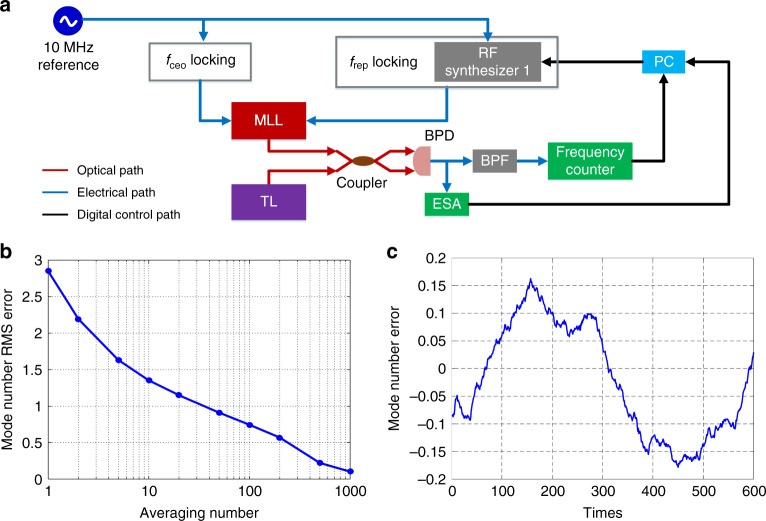
Self-calibration of the OFS. **a** During the calibration process, both *f*_ceo_ and *f*_rep_ of the MLL are locked to the 10 MHz reference. The beat note between the MLL and the free-running TL is traced by an electronic spectrum analyzer (ESA) and measured by a frequency counter. A computer (PC) is used to tune the frequency *f*_1_ within the *f*_rep_ locking block and collect the real-time results from the ESA and the frequency counter. **b** Mode number root-mean-square (RMS) error with respect to averaging numbers. **c** Mode number calibration error after 1000 times of averaging (*n* = 936, *M* = 766,746).

The mode number, *M*, can be decided by a calibration procedure^30^ with the TL-to-comb circuit unlocked. As shown in Fig. 3a, we use a computer to control the reference frequency, *f*_1_, within the *f*_rep_ locking circuit of the MLL in Fig. 2a so that the repetition rate of the MLL always follows a change in *f*_1_. We first continuously increase *f*_1_ while tracing the beat note, *f*_b_, between the TL and the MLL. When *f*_b_ has increasingly passed a specified frequency, *f*_b0_, *N* times, we record the instantaneous frequencies *f*_12_ and *f*_b2_. Subsequently, we continuously decrease *f*_1_. Once *f*_b_ has decreasingly passed *f*_b0_ *N* times, we record the instantaneous frequencies *f*_11_ and *f*_b1_. Then, the mode number can be calculated by:

2 $$M = \frac{{Nf_{12} + 4f_{\mathrm{b}2} - 4f_{\mathrm{b}1}}}{{f_{12} - f_{11}}}$$

The calibration error is mainly due to the thermal instability of the free-running TL. To reduce this error, a large value of 936 is chosen for *N*. Furthermore, we repeat the above procedures 1600 times and calculate the averaged *M* values using a digital-filter-like method. For example, a 100-time averaging level means taking the average from the 1st to 100th measurement, 2nd to 101st measurement, and so on, until the 1501st to 1600th measurement. The mode number RMS error decreases approximately with the square root of the averaging number, as shown in Fig. 3b. For an averaging level of 1000 times, the mode number error relative to the closest adjacent integer (766,746) is given in Fig. 3c. Since the error is well below 0.5 (<±0.2), *M* can be precisely determined.

### System tuning and instability

**Fig. 4:**
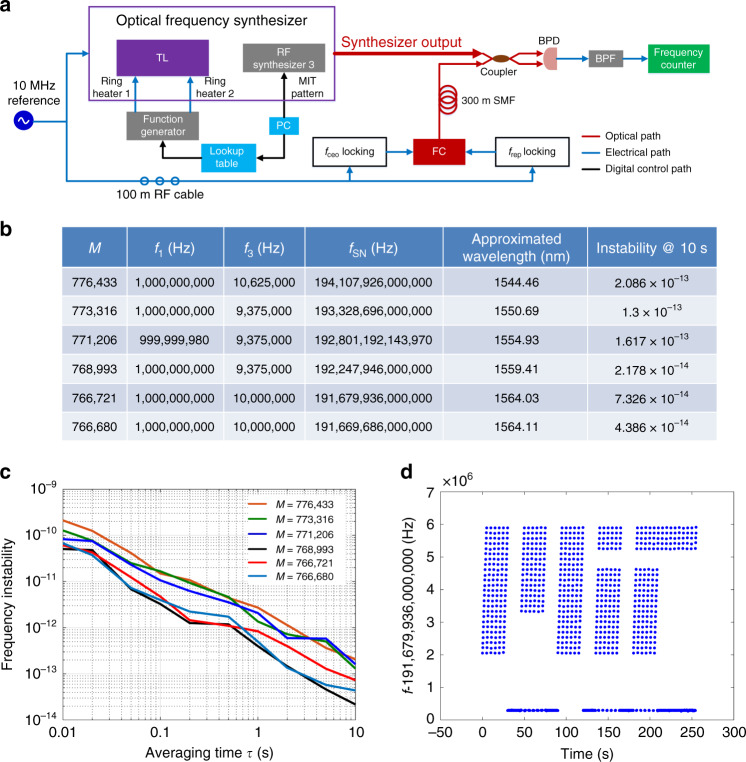
OFS characterization results. **a** The characterization setup. A frequency comb (FC), which is located in a different laboratory from the OFS, is used for an out-of-loop measurement. The carrier offset frequency and repetition rate of the FC are locked to the 10 MHz reference with a 100 m RF cable. The output of the FC beats with the synthesizer output after transmission through a 300 m single mode fiber (SMF). The beat note from the BPD is then filtered by a BPF and measured by a frequency counter. **b** The measurement results with six different *M* values. For all cases, *f*_2_ = 6.16 MHz. **c** Allan deviation for the six measured synthesizer frequencies across the C-band, showing a frequency instability of 10^−12^ at 1 s. **d** Fine frequency tuning to generate the MIT logo pattern (the averaging time of the frequency counter is 200 ms).

To evaluate the frequency instability of the OFS, we use the setup in Fig. 4a to perform an out-of-loop measurement. A full tuning range from 1544 nm (194.1 THz) to 1564 nm (191.6 THz) is achieved for the OFS by applying electrical power to the two microring heaters of the TL. For a tuning range larger than 2 nm, the approximate electrical power required by the two microring heaters can be obtained from a lookup table (see Materials and methods, large-range tuning), and a new calibration procedure is necessary before fine tuning the heaters’ power to set the OFS frequency to the exact target value. Five different wavelengths (1544.46, 1550.69, 1554.93, 1559.41, and 1564.03 nm) are chosen, and the calibrated *M* values are shown in the first five rows of Fig. 4b. Figure 4b also gives the final OFS output frequencies, *f*_SN_, and their corresponding *f*_1_ and *f*_3_ values in the experiments. By changing the electrical power of the two microring heaters simultaneously, we can continuously tune the OFS frequency relative to a calibrated value by up to ~2 nm (see Materials and methods, middle-range tuning). Using this method, the OFS frequency is decreased by 10.25 GHz (from 1564.03 nm), as given in the last row of Fig. 4b. Figure 4c shows the measured frequency instability (Allan deviation) of the six synthesizer frequencies obtained in Fig. 4b. For all cases, the frequency instability approximately drops inversely proportional to the averaging time τ. Figure 4c shows a frequency instability level of 10^−12^ at an averaging time of 1 s. At 10 s of averaging time, the frequency instability of each case ranges from 2 × 10^−13^ to 2 × 10^−14^ (last column of Fig. 4b).

For fine tuning at the sub-GHz level, the OFS frequency can be simply tuned by changing *f*_3_ in the TL-to-comb locking circuit to change the feedback electrical power to the TL phase shifters (see Materials and methods, small-range tuning). The RF synthesizer 3 can be programmed by a PC to generate an arbitrary pattern for the OFS output over time-frequency coordinates. Figure 4d provides an example of the MIT logo obtained from this fast and precise tuning mechanism.

## Discussion

Although the reported TL output power and slope efficiency are not high^29^, the power is sufficient for the OFS application. In the synthesizer setup, the saturation power of the BPD in Fig. 1 (Thorlabs PDB465C-AC) is 120 µW, which enables a >30 dB signal-to-noise ratio (SNR) for the electronic beat note after the BPD (using a 20 kHz resolution bandwidth on an electronic spectrum analyzer (ESA)) with <100 µW optical power from the TL. After the frequency divider in the TL-to-comb locking circuit (Fig. 2c), this SNR can be further improved to >80 dB, which is more than enough to obtain tight locking.

During the measurement shown in Fig. 3b, c, the repetition rate of the MLL needs to be tuned by ~300 kHz to achieve the value 936 for *N*. This process takes ~6 s because we need to move a motorized stage in the cavity of the MLL. Therefore, the total calibration time is ~3.3 h with 1000 times of averaging. In the future, by using an integrated MLL^31,32^, the repetition rate can be tuned by ~1 MHz within 1 ms using integrated heaters, so the overall calibration time can be reduced to <1 s.

In Fig. 4a, the voltage noise floor of the function generator is proportional to the output voltage range. In other words, a high-level output voltage *V*_H_ usually exhibits a large absolute RMS noise *V*_N_, which can be further enhanced through (*V*_H_ + *V*_N_)^2^, when we convert the voltage noise into the power noise. Thus, the higher the electrical power imposed on the two microring heaters, the more thermo-unstable the OFS is. In Fig. 4c, comparing two wavelengths of 1544.46 nm (*M* = 776 433) and 1559.41 nm (*M* = 768 993), the OFS requires much higher electrical power for operation in the first case; therefore, the frequency instability in the first case is almost 10 times higher than that in the latter case. The frequency instability performance can be improved by developing a low-noise voltage/power source with noise filters at the source output. With a better voltage/power source, it is also possible to tune the OFS within the full erbium gain bandwidth, showing good frequency instability performance.

In summary, we have demonstrated an OFS using a fully integrated erbium-doped TL on a silicon photonics platform. The synthesizer can be self-calibrated and supports a 2.5 THz (20 nm) tuning range. The typical frequency instability is 10^−13^ at a 10 s averaging time. Precise and flexible tuning capability is also demonstrated by generating an MIT logo pattern. Using the techniques in this work and other key components on a silicon photonics platform, including an octave-spanning supercontinuum generator^33^, second-harmonic generator^34^, and integrated MLLs^31,32^, there is great potential to build a completely monolithically integrated low-cost OFS in the near future.

## Materials and methods

### Numerical simulation

Effective indices and guided modes in waveguides are simulated using a vector finite-difference 2D eigenmode solver with a discretization of 20 nm. The code is written in MATLAB, and it solves the wave equation of the transverse electric field.

### Erbium TL fabrication

The TL is largely fabricated on a 300-mm silicon wafer in a state-of-the-art CMOS foundry at CNSE SUNY. The wafer has etched laser trenches for gain film deposition. After the wafer-scale fabrication, the wafer is diced, and an Al_2_O_3_:Er^3+^ gain film is deposited as a single-step back-end-of-line process at MIT, allowing direct access to the laser design^23^. The 1.1-µm-thick Al_2_O_3_:Er^3+^ thin film within the laser trench together with the trench sidewall provides the mode confinement for the gain waveguide. The deposition is performed by a reactive co-sputtering process using both aluminum and erbium targets. The substrate temperature during deposition was measured to be 380 °C. Deposition runs with different doping levels reveal an optimum Er^3+^ doping concentration of 1.5 × 10^20^ cm^−3^. Given the same pump power, a lower doping concentration will decrease the lasing power due to lower gain, while too high of a concentration will also result in lower lasing power due to severe ion clustering or quenching^35,36^. To keep the metal pads open after the Al_2_O_3_ thin film deposition process, the metal pads are placed in one area of the layout mask, and this area is covered by a glass plate during the Al_2_O_3_ thin film deposition. More details about the area selective deposition can be found in ref. ^37^.

### Large-range tuning

**Fig. 5:**
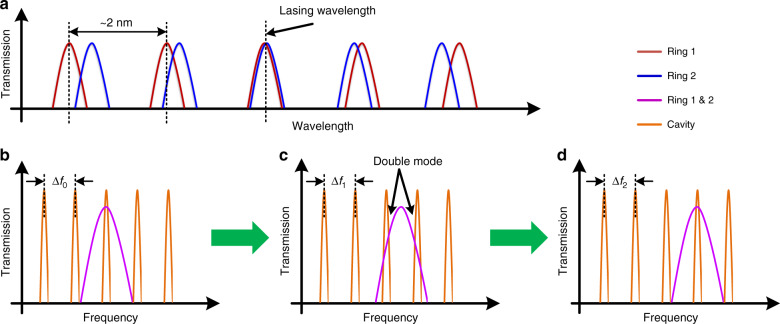
Frequency tuning mechanism of the TL. **a** Large-range tuning and **b**–**d** middle-range tuning.

There are two microring filters in the TL. Each ring has a periodic transmission spectrum (blue and red curves in Fig. 5a), with free spectral ranges (FSRs) of 2.23 and 2.13 nm at room temperature, respectively. This vernier configuration provides a combined FSR of 50 nm within the erbium gain bandwidth, which guarantees a single lasing wavelength of the TL. By changing the electrical power of the integrated heater on top of each ring, the FSR of each ring can be varied slightly; therefore, the TL will lase at a different wavelength where the new transmission spectra of the two rings overlap. For example, in Fig. 5a, the third red curve of ring 1 overlaps with the third blue of ring 2; if we change the voltage on the integrated heater of ring 2 to increase the FSR of ring 2, the fourth red curve may overlap with the fourth blue curve, thus achieving wavelength tuning. Based on this mechanism, a lookup table can be created to record all possible voltage combinations applied to the two ring heaters and their corresponding lasing wavelengths. In practice, with the help of the lookup table, by simply modifying the voltages of the ring heaters, an arbitrary output wavelength of the TL within the erbium gain bandwidth can be provided.

### Middle-range tuning

Different from the large-range tuning, if we change the voltages applied to the two ring heaters simultaneously, two specific transmission spectrum curves (e.g., the third red curve and the third blue curve in Fig. 5a) of the two rings can move synchronously; therefore, the combined ring response (magenta-color curves in Fig. 5b–d) can be continuously tuned without wavelength hopping. We can use the cavity transmission response of the TL (orange curves in Fig. 5b–d), which has an FSR of Δ*f* ≈ 2 GHz, to calculate the exact tuning frequency of the combined ring response. To maximize the TL output power, the peak of the combined ring response needs to overlap with one peak of the cavity response, as shown in Fig. 5b. When we synchronously change the voltages on the two ring heaters, the combined ring response can be tuned from the position in Fig. 5b to that in Fig. 5c, d. At the same time, the cavity response FSR, Δ*f*, is also slightly changing (from Δ*f*_0_ to Δ*f*_1_ to Δ*f*_2_) because of the optical length change of the two rings. When the combined ring response is in the middle of two cavity response peaks, as shown in Fig. 5c, the TL can output two longitudinal modes, which can generate four beat notes *f*_*d*1_, *f*_rep_ − *f*_*d*1_, *f*_*d*2_, and *f*_rep_ − *f*_*d*2_ in [0, *f*_rep_] after beating with an MLL, where *f*_rep_ is the repetition rate of the MLL. Suppose *f*_*d*1_ and *f*_*d*2_ decrease with increasing ring heater voltage; then, the current cavity response FSR, Δ*f*_new_, can be calculated by

3 $$\Delta f_{\mathrm{new}}{\mathrm{ = }}\left[ {\frac{{\Delta f_{\mathrm{est}} + f_{d1} - f_{d2}}}{{f_{\mathrm{rep}}}}} \right]_{{\mathop{\mathrm{int}}} }f_{\mathrm{rep}} + f_{d2} - f_{d1},$$

where Δ*f*_est_ is the estimated FSR value from an earlier calculation or measurement, and the square bracket “[*x*]_int_” returns the integer closest to *x*. In practice, Δ*f* can be initially estimated from the design parameters or measured from the self-beating frequency of the TL by setting the device into a double-longitudinal-mode state with little ring heater power. Then, by synchronously increasing the voltages applied to the two ring heaters and tracing the beat notes between the TL and MLL on an ESA, Δ*f* can be iteratively updated by Eq. 3. Therefore, the frequency of the TL can be accurately tuned across many cavity longitudinal modes. Theoretically, the laser can be tuned over the full erbium gain bandwidth using this middle-range tuning mechanism. However, in practice, we cannot unlimitedly increase the heater voltage without breaking the heater, and if the tuning range is larger than one FSR of the ring response (~2 nm), we can already use the large-range tuning mechanism, so one FSR of the ring response is chosen as the maximum tuning range of the middle-range tuning.

### Small-range tuning

There are two longitudinal-mode phase shifters in the TL. The heaters for the two phase shifters are controlled by the same voltage. By varying this voltage, the frequency of TL can be continuously tuned within one FSR of the cavity transmission response, that is, ~2 GHz.
